# Porcine liver injury model to assess tantalum-containing bioactive glass powders for hemostasis

**DOI:** 10.1007/s10856-022-06674-3

**Published:** 2022-06-07

**Authors:** Malvika Nagrath, Danielle Bince, Corwyn Rowsell, Deanna Polintan, Joao Rezende-Neto, Mark Towler

**Affiliations:** 1 Biomedical Engineering, Faculty of Engineering and Architectural Science (FEAS), Ryerson University, Toronto, M5B 2K3 ON Canada; 2 Li Ka Shing Knowledge Institute, St. Michael’s Hospital, Toronto, M5B 1W8 ON Canada; 3 Research Vivarium, St. Michael’s Hospital, Toronto, M5B 1W8 ON Canada; 4 Department of Laboratory Medicine, St. Michael’s Hospital, Toronto, M5B 1W8 ON Canada; 5 Department of Laboratory Medicine and Pathobiology, University of Toronto, Toronto, M5S 1A1 ON Canada; 6 Trauma and Acute Care, General Surgery, St. Michael’s Hospital, Toronto, M5B 1W8 ON Canada; 7 Department of Surgery, University of Toronto, Toronto, M5S 1A1 ON Canada; 8 Department of Mechanical and Industrial Engineering, FEAS, Ryerson University, Toronto, M5B 2K3 ON Canada

## Abstract

This study evaluates compositions of tantalum-containing mesoporous bioactive glass (Ta-MBG) powders using a porcine fatal liver injury model. The powders based on (80-*x*)SiO_2_-15CaO-5P_2_O_5_-*x*Ta_2_O_5_ compositions with *x* = 0 (0Ta/Ta-free), 1 (1Ta), and 5 (5Ta) mol% were made using a sol–gel process. A class IV hemorrhage condition was simulated on the animals; hemodynamic data and biochemical analysis confirmed the life-threatening condition. Ta-MBGs were able to stop the bleeding within 10 min of their application while the bleeds in the absence of any intervention or in the presence of a commercial agent, Arista^TM^ (Bard Davol Inc., Rhode Island, USA) continued for up to 45 min. Scanning electron microscopy (SEM) imaging of the blood clots showed that the presence of Ta-MBGs did not affect clot morphology. Rather, the connections seen between fibrin fibers of the blood clot and Ta-MBG powders point towards the powders’ surfaces embracing fibrin. Histopathological analysis of the liver tissue showed 5Ta as the only composition reducing parenchymal hemorrhage and necrosis extent of the tissue after their application. Additionally, 5Ta was also able to form an adherent clot in worst-case scenario bleeding where no adherent clot was seen before the powder was applied. In vivo results from the present study agree with in vitro results of the previous study that 5Ta was the best Ta-MBG composition for hemostatic purposes.

**Figure:**
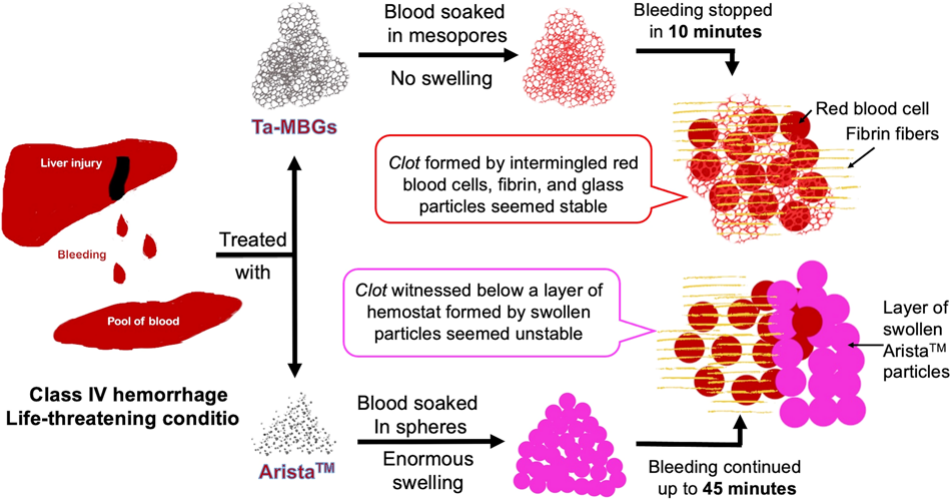
Graphical abstract

Graphical abstract

## Introduction

Bioactive glasses (BGs) are usually applied as bone fillers [1, 2]. BGs degrade in the biological environment releasing therapeutic ions that lead to the formation of hydroxyapatite crystals; the inorganic component of the bone [1, 3, 4]. BG-induced osteogenesis is dependent on factors such as pH, chemistry, and release of the ions from the glasses themselves [1–4]. BGs have also been tried in the field of hemostasis [5–16], where, along with the release of ions and the chemical composition of BGs, certain structural features (mesopores and high surface area) play an essential role [5–16].

Hemorrhage is the leading cause of death on the battlefield and the second most common cause of civilian mortality following trauma [17–20]. It is also the cause of most operating room deaths, post-trauma [17, 18, 21]. Hemorrhage may also be complicated with hypothermia, acidosis, and dilutional coagulopathy due to excessive blood loss, reduction in clotting factors, and clotting factor activity; the combination of those conditions is also known as the lethal triad [19–22]. About 25% of severe trauma cases result in coagulopathy increasing mortality rate four times [19–21, 23]. The liver is the most commonly injured solid organ in the abdominal cavity in both blunt and penetrating traumas [17, 18, 20, 24]. Accordingly, porcine hemorrhagic shock models are frequently used to investigate hemorrhage-related coagulopathy [17–22, 24, 25].

Hemostasis is the body’s physiological attempt to stop bleeding [14, 26]. It is achieved through the mechanisms of vascular constriction, platelet plug formation, and coagulation [14, 26]. Coagulation is a complicated enzymatic process that is initiated by intrinsic and extrinsic pathways [14, 26]. These converge to a common pathway, leading to a stable blood clot [14, 26]. In surgical interventions, hemostatic dressings (hemostats) can help to achieve hemostasis [27, 28]. Various biomaterial-based hemostats are available commercially [29–33] and are being developed to enhance hemostasis [8–11, 13, 34, 35]. Hemostats in powder form are gaining popularity among surgeons due to their ease of use [36]. However, such powders can control only low levels of bleeding.[36, 37] Arista^TM^ (Bard Davol Inc., Rhode Island, USA), a purified starch product, is one of the popular powdered hemostats commercially available; but it has limitations [27, 36, 37]. Arista^TM^ shows 500% swelling after coming in contact with fluids; this can cause pressure on the tissues in the enclosed wounds leading to ischemia and hence necrosis [27, 32, 36, 37]. Arista^TM^ forms unstable clots in severe bleeding cases and needs multiple re-applications [37]. The need for a powder-based hemostat that can address these concerns and provide adequate hemostasis exists and BG powders are excellent candidates for these purposes.

BG-based enhancement of coagulation is generally due to the activation of the intrinsic pathway which is initiated by the activation of factor XII (FXII) [5, 6, 10, 12, 14, 34, 35, 38–42]. The negatively-charged surfaces, such as those provided by the BGs, can activate FXII leading to the augmentation of the intrinsic coagulation cascade and hence hemostasis [5, 6, 10, 12, 14, 34, 35, 38–42]. Mesoporous bioactive glasses (MBGs), a subset of BGs, fabricated using a sol–gel process, have been researched to amplify the process of hemostasis by boosting the clotting process [10, 12–14, 34, 35, 42]. MBGs possess high surface area due to the presence of mesopores in their structure which helps to absorb the fluid component of the blood, bringing the clotting factors in proximity [10, 12–14, 34, 35, 42]. This initiates the coagulation process. The presence of mesopores (within the glass structure) and voids between the glass particles provide capillary action, which helps to soak in the fluid component of the blood [34]. Additionally, protein adsorption at the biomaterial’s surfaces initiates biological events [43–45], and this protein adsorption is dependent on the surface chemistry and topography [43, 44]. The higher surface area of MBGs exposes higher Si-OH groups (surface chemistry effect) providing more negatively charged surfaces, and MBG’s nanoporous architecture (topography effect) provides a better matrix for protein adsorption for the initiation of biological activities [46]. BGs present negatively-charged surfaces due to silanol groups, which activate FXII [5, 13] accelerating the coagulation cascade. BGs can also provide calcium ions, Ca^2+^ (clotting factor IV), to the bleeding site [5, 13]. Ca^2+^ plays a ubiquitous role in the coagulation cascade, acting as a cofactor in the enzymatic reactions involved in the process of coagulation [13, 14, 26].

Silica-based ternary BG compositions (silicon–calcium–phosphorus, Si–Ca–P) have been explored for hemostasis [11, 34, 41, 42] because silanol groups can provide negative surfaces, and Ca^2+^ helps to amplify the coagulation process. The composition of the BGs can be customized with dopants [8–10, 35]: network modifiers can be incorporated in the glass compositions to achieve targeted applications [3, 4]. For example, *strontium* can be added to enhance in vivo bone formation, *zinc* for anti-inflammatory and antibacterial properties, *calcium* for hemostasis, and *gallium* for antibacterial, anti-inflammatory, and hemostatic capabilities [3, 4, 13]. *Tantalum* (Ta) shows an antibacterial, anti-inflammatory, and hemostatic potential [47–51]; for these reasons, Ta has been incorporated into the silicon–calcium–phosphorus (Si–Ca–P) MBG composition by the authors previously [52, 53]. Ta was incorporated in various amounts (0- *0Ta*, 0.5- *0.5Ta*, 1- *1Ta*, 5- *5Ta*, and 10- *10Ta* mol%) to evaluate its hemostatic effectiveness [52, 53]. Tantalum-containing MBG (Ta-MBG) powders, fabricated using a sol–gel process, showed superior hemostatic potential to the Ta-free MBG compositions [52]. Mouse tail-cut models showed a ≥50% reduction in bleeding times with Ta-MBGs than Ta-free MBGs, Arista^TM^ and no treatment [52]. In-vitro testing concluded that 1Ta and 5Ta samples showed a better hemostatic potential among other Ta-MBGs [53].

In the present study, an acute porcine liver injury model representing a life-threatening condition with class IV hemorrhage was designed to ascertain the best hemostatic Ta-MBG composition. This in-vivo model was chosen because the liver is the most commonly injured organ in abdominal trauma and bleeding from severe liver injury is a major cause of death [17, 18, 20, 24]. This model simulates a worst-case scenario of traumatic liver hemorrhage. The current study presents the evaluation and comparison of two Ta-MBG powders, 1Ta and 5Ta, to Ta-free (0Ta) powder and a hemostatic commercial product in powder form, Arista^TM^.

## Materials and methods

### Materials

Reagent grade triblock copolymer P123 Poly(ethylene glycol)-block-poly(propylene glycol)-block-Poly(ethylene glycol), calcium nitrate tetrahydrate (≥99.0%), triethyl phosphate (≥99.8%, TEP), tetraethyl orthosilicate (98%, TEOS), tantalum (V) ethoxide (99.98%), reagent grade hydrochloric acid (HCl) and ethanol were purchased from Sigma Aldrich (Oakville, Canada). HCl was diluted to 0.5 N using distilled water.

### MBG fabrication method

Ta was incorporated in a (80-*x*)SiO_2_-15CaO-5P_2_O_5_-*x*Ta_2_O_5_ composition with *x* = 0 (0Ta/Ta-free), *x* = 1 (1Ta), and *x* = 5 (5Ta) mol%. The MBGs were synthesized using a sol–gel process by a method described in our previous publications.[52, 53] In short, 1 ml of HCl was pipetted into the appropriate amount of TEOS; the mixture was kept aside to initiate hydrolysis. In another beaker, 4 gm of P123 and 1.4 gm of calcium nitrate tetrahydrate were dissolved in 76 ml of ethanol; the mixture was kept on the stirrer. To the beaker on the stirrer, 0.68 ml of TEP and *x* mol% of tantalum (V) ethoxide were added; and lastly, TEOS-HCl solution was added to the mixture. After the mixture’s overnight stirring, a clear solution was formed and was transferred to the petri-dish for the evaporation-induced self-assembly (EISA) process. EISA converted the sol to the gel form in about a week. The derived gel underwent a thermal process at 650 °C for 6 h at a heating rate of 1 °C/min for calcination. The calcined glass was ground and sieved through a 45 μm mesh to collect Ta-MBGs. All the further experimentation was performed on these fabricated Ta-MBGs.

### Surgical procedure

**Fig. 1:**
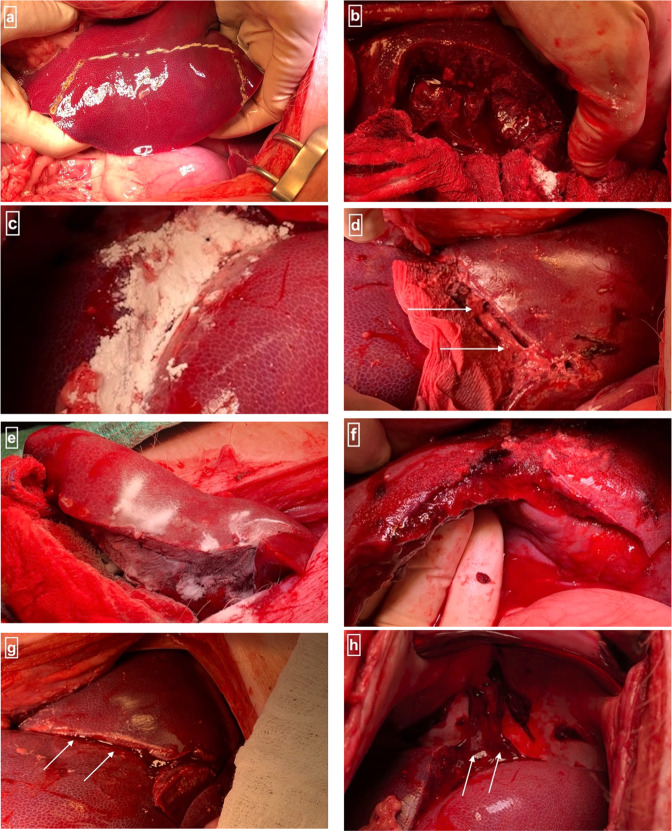
In vivo porcine liver injury and intervention application. This shows the representative images of the porcine liver injury in vivo surgery: **a** the part of the liver is marked for avulsion; **b** the injured section of the liver is shown from which the liver tissue was avulsed; **c** the sprinkled Ta-MBG powder; **d** the bleeding site with Ta-MBG at 5 min time point; **e** sprinkled Arista^TM^ powder; **f** the bleeding site with Arista^TM^ at 5 min time point; **g** the bleeding site with Ta-MBG at 10 min time point; folding of the liver is evident here (arrows); **h** blood clot (arrows) at the injured site with Ta-MBG

The St. Michael’s Hospital Animal Care Committee approved the porcine liver injury protocol (Approved protocol #726). The animal trials comply with the National Institutes of Health guide for the care and use of Laboratory animals (NIH Publication No. 8023, revised 1978). 40–60 kg weighing male Yorkshire porcine were acquired from Caughell Farms, Fingal ON, Canada, and housed in the animal facility at Li Ka Shing Knowledge Institute, St. Michael’s Hospital, Toronto. The porcine was anesthetized using a premedication of Ketamine (20 mg/kg) + Xylazine (2 mg/kg) + Atropine Sulfate (1 mg/25 kg) followed by inhalation of 2–5% Isoflurane (10 ml/kg) for induction and maintenance of the anesthesia. Once anesthetized, the animals were intubated and maintained on a ventilator at 2–3% Isoflurane (10 ml/kg) for the duration of the procedure. Animals were monitored via jaw tone, pulse oximetry and Electrocardiogram, temperature, End-tidal CO_2_, Intra-arterial Blood Pressure, and Central Venous Pressure. A laparotomy was performed to expose the liver; the spleen was removed to prevent autotransfusion. Severe hemorrhage began with removal of 35% of the animal’s total blood volume through a catheter placed in the left carotid artery. Coagulopathy was created by placing ice packs in the abdominal cavity (35–36 °C). The animal’s resuscitation was initiated after 20 min using 1000 ml of warmed intravenous lactated ringer’s solution to maintain the mean arterial pressure at 60 mmHg. Hepatic injuries (Fig. 1a and b) were performed using a #11 surgical blade. A 3 × 10 cm rectangular segment of the right middle lobe of the liver was removed (Fig. 1b). Subsequently, free bleeding from the injury site was allowed for two minutes to simulate uncontrolled bleeding and exacerbate hemorrhagic shock. The animals lost ~450 ml of blood during the free bleeding phase and up to 1100 ml of blood until the end of the experiment, simulating a class IV hemorrhagic shock. Hemorrhage control from the liver injury was obtained using one of the five interventions (Fig. 1c):

- Gauze packing group (*n* = 2): only gauzes
- 0Ta group (*n* = 2): 0Ta (Ta-free) MBG powder with the gauze
- 1Ta group (*n* = 2): 1Ta MBG powder with the gauze
- 5Ta group (*n* = 2): 5Ta MBG powder with the gauze
  - 1Ta and 5Ta groups together constituted Ta-MBG group
- Arista^TM^ group (*n* = 2): Commercial hemostat Arista^TM^ with the gauze

The powder samples were sprinkled over the bleeding sites (Fig. 1c). After depositing approximately1g over the active bleeding, the site was compressed using gauze for five minutes. Then, the gauze was lifted to visualize the need for more hemostat (experimental intervention) and additional powder (~1–2 g) was placed if necessary. The bleeding was checked after another 5 min. At the end of the procedure, the pigs were euthanized by an overdose of anesthesia followed by an injection of T61 veterinary euthanasia solution.

### Analytical tests

The following data were collected to analyze the liver injury model:

#### Blood tests

Arterial blood samples were collected at baseline and the end of the procedure for the blood work: coagulation profile.

#### Bleeding time

The time to cease the bleeding was noted. Bleeding times were broadly classified as within 5 min, 5–10 min, and beyond 10 min for the present study.

#### Blood clot imaging

Blood clots with and without Ta-MBGs were collected for Scanning Electron Microscopy (SEM) analysis. The collected blood clots were fixed using 2.5% glutaraldehyde for a minimum of 24 h, followed by post-fixing in 1% osmium chloride for 1 h. Samples were washed and underwent a graded dehydration series of ethanol and HMDS (hexamethyldisilazane) drying for 10 min each. The clots were left in the fume-hood to dry for 24 h, followed by gold sputtering before imaging the clots with SEM. The samples were imaged using a scanning electron microscope (SU3500, Hitachi, Japan) at an accelerating voltage of 5.00 kV.

#### Histopathological studies

**Fig. 2:**
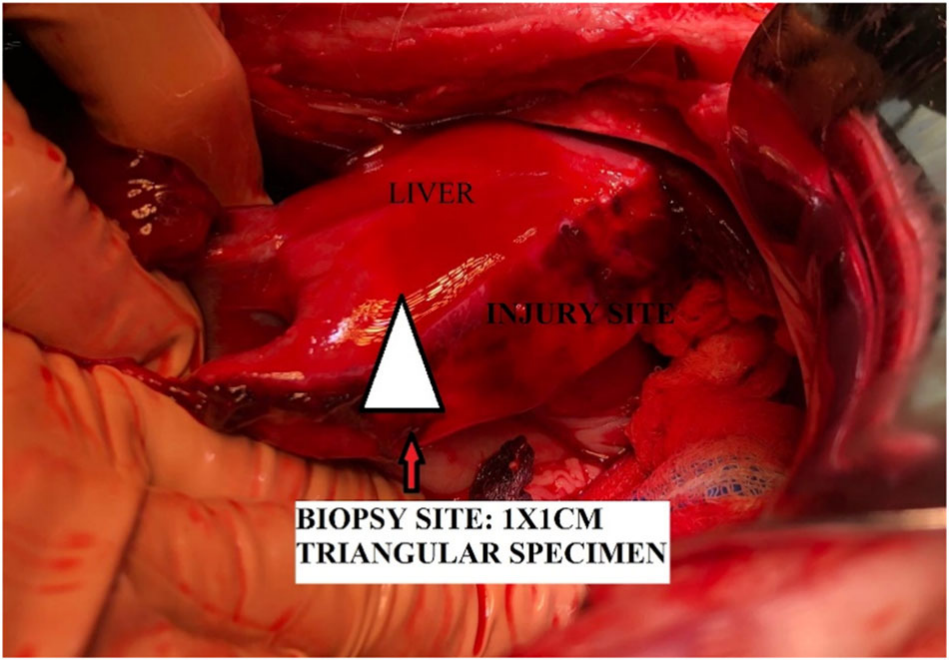
This shows the representative site from where liver tissue was taken for histopathology

Were performed to compare liver tissue in the presence and absence of hemostatic interventions (*n* = 1). 1 × 1 cm liver tissue pieces (with and without samples) were collected and preserved in 10% formaldehyde for histopathological analysis. The triangular tissue specimen was always taken from the same site, as shown in Fig. 2:

The fixed samples were labeled to blind the pathologist to the interventions used in the study. After at least 24 h of fixation in 10% formaldehyde, tissue samples were processed on a Leica Peloris II Tissue Processor (Rankin, MI, USA) and paraffin-embedded. Tissue sections of 4-micron thickness were cut and placed on 75 mm × 25 mm glass slides and stained with hematoxylin and eosin (H&E) stains on a Leica HistoCore SPECTRA ST automated Stainer (Lecia Biosystems, ON, Canada). The H&E slides were assessed on a Leica DM 2500 bright-field optical microscope (Lecia Microsystems, Wetzlar, Germany). The prepared slides were evaluated to collect semi-quantitative histopathological analysis based on the following parameters: (a) *hemorrhage content* (the greatest depth of parenchymal hemorrhage relative to the injury site, measured in mm), (b) *hemorrhage percent* (the percentage of liver tissue with parenchymal hemorrhage), (c) *necrosis extent* (the greatest depth of necrosis relative to the injury site, measured in mm), and (d) *adherence of clot to the injured site* (qualitative parameter defined in Table 1).

**Table 1:** Description of the adherent clot scale

Adherent clot scale	Description
**−**	None seen
**+**	Minimal/mild amount of adherent clot
**++**	Moderate amount of adherent clot
**+++**	Extensive adherent clot

## Results

### Blood tests

The results of baseline and end of the procedure blood tests, coagulation profile are given in Tables 2 and 3. Table 2 Coagulation profile (Table 2) denotes the effect of experimental groups on the bleeding animals’ coagulation ability.

**Table 2:** Coagulation profile (time ± standard error of mean)

	Baseline			End of the procedure		
	Fibrinogen (g/L)	PT (s)	PTT (s)	Fibrinogen (g/L)	PT (s)	PTT (s)
Gauze group (*n* = 1)	1.1	15.8	11.1	0.6	19.2	13.6
Ta-free group (*n* = 1)	1.3	16.1	15.9	0.7	18.4	16.9
Ta-MBG group (*n* = 2)	1.6 ± 0.05	14.4 ± 0.6	13.3 ± 1.5	1 ± 0.1	15.5 ± 0.3	12.8 ± 0.8
Arista^TM^ group (*n* = 2)	1.2 ± 0.2	16.9 ± 0.9	17.4 ± 0.5	0.8 ± 0.3	18.1 ± 0.3	13.7 ± 0.9

**Table 3:** Bleeding times for all the groups

	Bleeding times (min)
Gauze group (*n* = 2)	Beyond 10
Ta-free group (*n* = 2)	5–10
1Ta group (*n* = 1)	5–10
5Ta group (*n* = 2)	Within 5
Arista^TM^ group (*n* = 2)	Beyond 10

For all groups, excessive blood loss led to reduced fibrinogen levels at the end of the procedure compared to baseline (Table 2). Additionally, PT increased for all the groups while PTT decreased (except for the gauze and 0Ta groups) at the end of the procedure than baseline (Table 2).

### Bleeding times

5Ta samples ceased the bleeding within 5 min of its application while 0Ta and 1Ta stopped the bleeding between 5 and 10 min (Table 3). However, Arista^TM^ and gauze groups did not stop the bleeding within 10 min (Table 3); continuous bleeding was seen even at 45 min for Arista^TM^ and gauze groups.

### Blood clot imaging

**Fig. 3:**
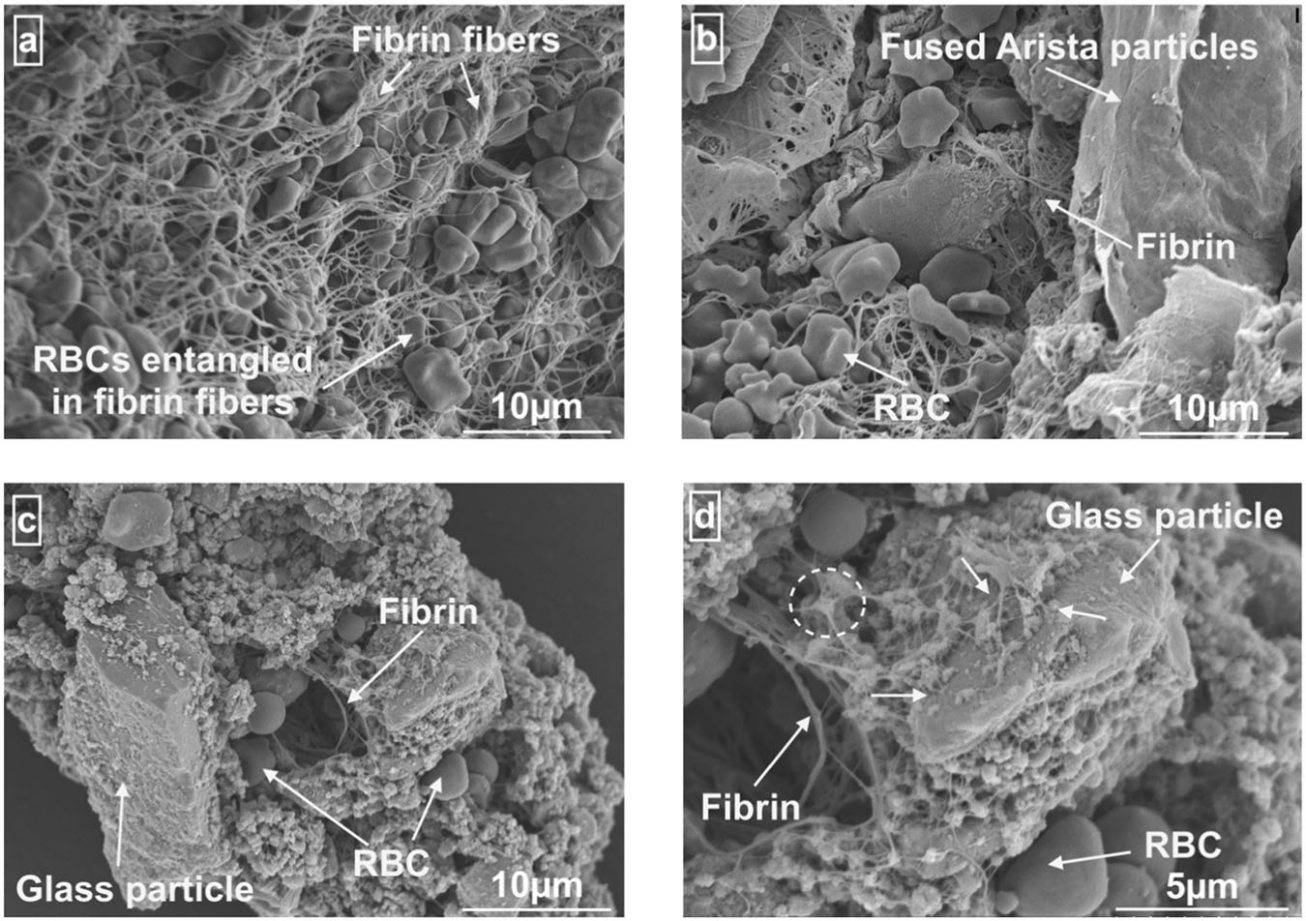
Blood clot evaluation. It shows SEM images of the blood clots: **a** *clot without a sample*: a physiological blood clot shows RBCs entrapped in the fibrin meshwork; **b** *clot with Arista*^*TM*^: the Arista^TM^ particles are fused to form a flat sheet below which a blood clot (RBCs entangled in the fibrin meshwork) is seen, **c** *clot with Ta-MBG*: a blood clot is seen around Ta-MBG particles and glass particles are seen to agglomerate with the RBCs and fibrin meshwork; and **d** *high-resolution image of blood clot with Ta-MBG*: the arrows represent the area where fibrin network is seen to connect with the glass particles; the area marked by a dotted circle shows a glass particle from which multiple fibrin fibers seem to originate

Figure 3a and c represents SEM images of the blood clot in the absence and presence of Ta-MBG samples, respectively. Both the images show RBCs entangled in the fibrin meshwork. Ta-MBGs did not show any adverse effect on clot morphology. On further evaluation of the blood clot in the presence of Ta-MBGs at a higher resolution (Fig. 3d), a connection between the fibrin network and glass particles is evident. However, Arista^TM^ particles are seen to fuse to form a flat sheet and the RBCs entangled in the fibrin network can be seen below the sheet (Fig. 3b).

### Histopathological analysis

**Fig. 4:**
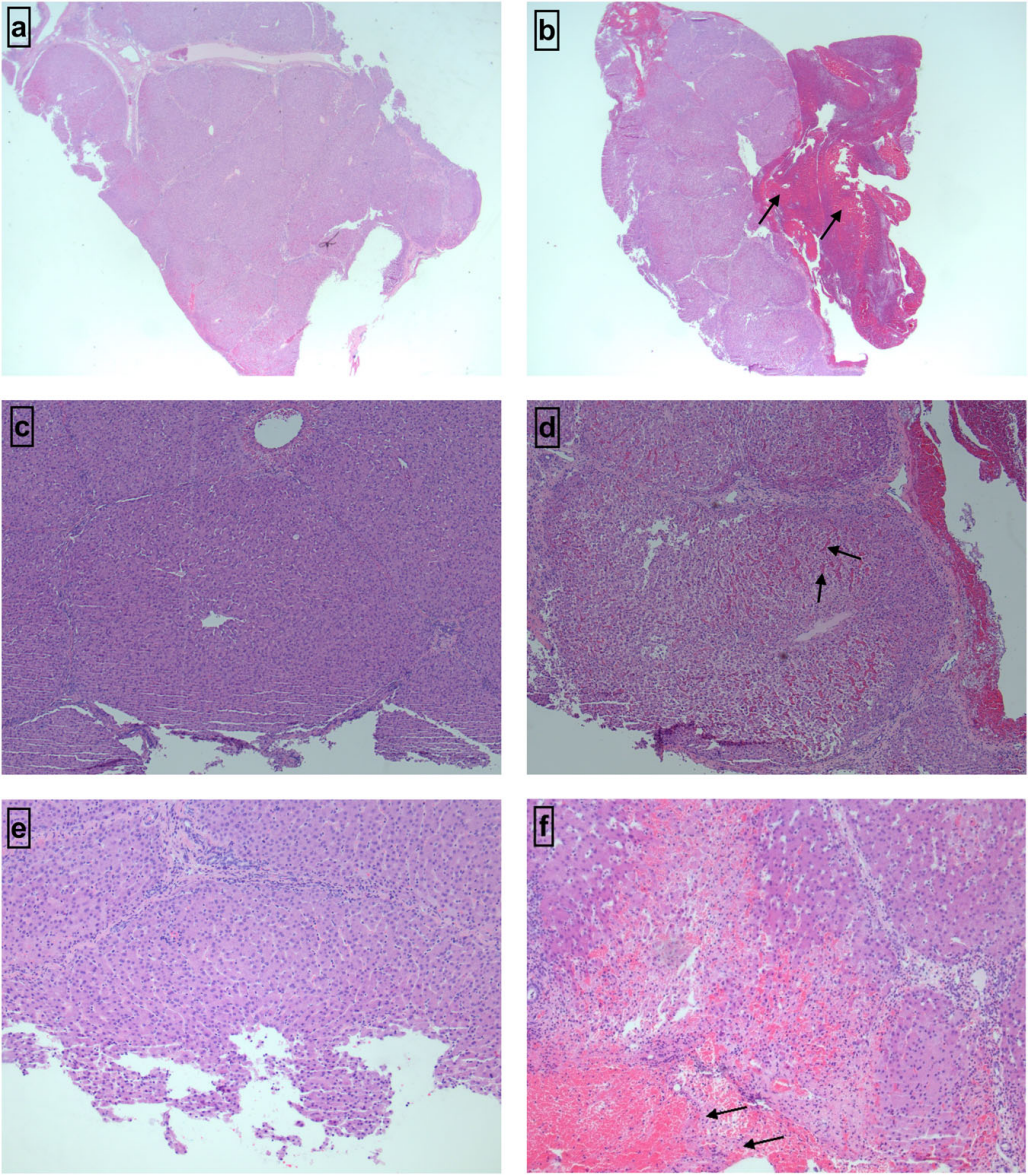
Histopathological evaluation. Histology images of the liver tissue: **a** and **b** show the presence and absence of adherent clots at 10×; **c** and **d** show hemorrhage extent at 50×; **e** and **f** show the necrosis extent at 100×. **a** Liver tissue displays no adherent clot (−) before the intervention; no dark reddish stains representing adherent clots are seen. **b** Liver tissue shows extensive adherent clot (+++) following hemostatic interventions; abundant dark reddish stained tissue (arrows) means adherent clot. **c** Liver tissue does not show any bleeding at the start of the procedure; **d** Liver parenchyma shows 60% hemorrhage with an extent of 1.76 mm; redness in the interstitial parenchyma (arrows) is representative of the bleeding. **e** Liver tissue does not show any necrosis at the start of the procedure; **f** represents a maximum necrosis extent of the liver tissue seen in the study; light pink stained tissue (arrows) represents the necrotic areas while red-stained area shows hemorrhage associated with the necrosis

Table 4 shows the semi-quantitative histopathological grading of the liver tissue before and after the intervention for the same animal. The clot adherence increases after applying intervention (Ta-MBGs and commercial hemostat, Arista^TM^). Except for 5Ta, the hemorrhage extent, hemorrhage percent, and necrosis extent of the tissue increase after applying the intervention. Figure 4 shows the H&E images of the adherent clot, hemorrhage extent, hemorrhage percent, and necrosis extent seen histologically before and after intervention in the present study.

**Table 4:** Semi-quantitative histopathological grading of the liver tissue

		Adherent clot	Hemorrhage extent (mm)	Hemorrhage percent (%)	Necrosis extent (mm)
**Ta-free** (*n* = 1)	*Before*	**+**	0	0	0
	*After*	**+++**	1.76	60	0.4
**1Ta** (*n* = 1)	*Before*	**+**	0	0	0
	*After*	**+++**	1.12	5	0.24
**5Ta** (*n* = 1)	*Before*	**−**	1.68	10	0.88
	*After*	**+**	0.8	5	0.64
**Arista**^**TM**^ (*n* = 1)	*Before*	**+**	0	0	0
	*After*	**+++**	0.96	5	0.24

## Discussion

The hemorrhagic shock model described in this study replicates the clinical findings that lead to dilutional coagulopathy post-resuscitation due to the loss of clotting factors especially *fibrinogen* [22, 54]. Fibrinogen is an essential clotting protein that gets converted to fibrin fibers to provide the meshwork to form a stable clot [14, 26]; a native conformation of fibrinogen is vital for its conversion to fibrin fibers [14, 26]. A fibrinogen level below 1.5 g/L can cause bleeding complications [22, 54]. Multiple studies [22, 25] have demonstrated that managing fibrinogen deficiency in a severe porcine liver injury model with dilutional coagulopathy leads to a reduction in blood loss and clot formation time. In the present study, excessive blood loss led to reduced fibrinogen levels between 0.6 and 1 g/L (Table 2), capable of causing bleeding complications. Despite the reduced fibrinogen level, Ta-MBGs could stop the bleeding within 10 min of their application. Roach et al. [44] suggested that silica spheres less than 30 nm diameter led to a high conformational distortion of fibrinogen, affecting its function. Ta-MBGs are 45 µm in size [52] and do not seem to affect fibrinogen’s function in the present study. In contrast, gauze and Arista^TM^ groups could not stop hemorrhage at the end of 10 min; bleeding was evident even at 45 min.

PT and PTT are standard clinical tests used by physicians to analyze the patients’ coagulation abilities [26]. BG-induced coagulation is achieved by the enhanced activation of the intrinsic coagulation cascade, which is assessed using PTT, while PT evaluates the extrinsic pathway of coagulation [8, 9, 11, 13, 35, 53]. As expected (except for Arista^TM^), all experimental groups showed an increase in PT at the end of the procedure. Even though previous publications showed that Arista^TM^ enhances the extrinsic pathway of coagulation [27], it did not reduce PT in our study; instead, it reduced PTT, which measures the activity of the intrinsic coagulation cascade. Ta-MBGs and Arista^TM^ reduced PTT while gauze and Ta-free MBG groups raised PTT at the end of the procedure (Table 2). This observation points to the role played by Ta-MBGs and Arista^TM^ to activate intrinsic coagulation pathways. The negatively charged surfaces enhance the activation of FXII, which initiates intrinsic coagulation [5, 13]. Our in vivo findings are in keeping with previous in vitro reports that showed enhanced activation of intrinsic coagulation pathway by Ta-MBGs with a reduction in PTT values [53] and bleeding times [52]. It is believed that the negatively-charged surfaces of Ta-MBGs (assessed using zeta potential values previously [53]) could activate intrinsic coagulation cascade as seen through in vitro and in vivo PTT measurements.

Ta-MBGs showed better hemostatic efficacy than Arista^TM^ in the severe hemorrhage model described in the present study. Ta-MBGs reduced PTT and bleeding times while Arista^TM^ displayed lower PTT but could not stop the bleeding even within 45 min. These observations can be explained by the difference in the mechanism of action by which Arista^TM^ and Ta-MBGs control bleeding. Arista^TM^, epichlorohydrin cross-linked purified potato starch, provides a porous surface for the absorption of fluid components of the blood, concentrating the clotting factors and hence inducing platelet plug formation [32, 36, 37, 55]. Essentially, Arista^TM^ provides a matrix to concentrate clotting factors and blood clot formation; it does not activate the coagulation cascade in any way [27, 36, 37]. On the contrary, Ta-MBGs, along with soaking the blood’s fluid component and providing a matrix to concentrate clotting factors and blood clot formation, also provide a negatively-charged surface to activate the intrinsic pathway of coagulation swiftly. The provision of negatively-charged surfaces (to trigger the coagulation cascade), in addition to concentrating clotting factors, elucidates Ta-MBGs superior hemostatic efficacy than Arista^TM^.

The literature suggests that rapidly degrading hemostats (such as Arista^TM^) are more suitable for managing low-level bleeds because rapid degeneration of Arista^TM^ leads to an unstable clot in cases of severe bleed [37]. Singh et al. [36] hypothesized Arista^TM^ forms a mechanical barrier to block the blood flow; the sufficient pressure built up below the barricade dislodges the blood clot, leading to re-bleeding. Multiple studies have reported the inadequate hemostatic effect of Arista^TM^ in severe bleeding models, such as the one described herein [37, 56, 57]. The literature also reports multiple re-applications of Arista^TM^ (up to 10 g) in an attempt to control profuse bleeding situations [37]. Similar conditions were observed in the present study; Arista^TM^ could not form a stable clot in a profuse bleeding condition. Visual differences between the clots formed by Ta-MBG (after 5 min of application) and Arista^TM^ (after 45 min of application) were appreciated. Ta-MBG appeared to provide a stable matrix to populate clotting factors for clot formation, while Arista^TM^ appeared to form an unstable surface with active bleeding sites. These observations are confirmed by the electron microscopy images of the blood clots. Singh et al. [36] described that Arista^TM^ forms a coalesced layer (which acts as a barrier to stop the bleeding) after soaking the fluids; such a fused film of Arista^TM^ was also seen in the present study. The physiological clot seems to be developing below the coalesced layer with no interaction with Arista^TM^ particles. Conversely, Ta-MBGs are seen to form an agglomerate with RBCs and fibrin fibers without disrupting normal clot morphology. A connection between the glass particles and fibrin fibers is also evident. The literature suggests that fibrinogen adsorption on the MBG surfaces is related to the nanopore size, Columbic forces between fibrinogen and glass, and hydrophobicity of the glass [46]. Lin et al. [46] suggested that fibrinogen penetration is inhibited in the nanopores smaller than 2 nm. Ta-MBGs used in the present study possess nanopores of about 4 nm [52] and probably were accessible to fibrinogen. Another study [58] explored the effect of mesopore size on the fibrinogen adsorption and hence the fibrin network formation. Considering fibrinogen’s dimensions: length of 47.5 nm (E domain) and diameter of 6 × 9 nm (D domain), they tested the effect of various mesopore sizes (2, 8, 14, and 20 nm) on the fibrinogen adsorption to analyze linear growth of fibrinogen’s D domain in clot formation [58]. Their study concluded that the pore sizes more than 9 nm (D domain dimension), i.e. 14 and 20 nm, were able to adsorb fibrinogen while the smaller mesopores (2 and 8 nm) had little effect [58]. Mesoporous architecture with 2 and 8 nm pores was instrumental in water absorption and hence the enhancement of clot formation, but the enhancement due to fibrinogen adsorption was minimal [58]. We hypothesize that there is a connection between fibrin and glass particles in our study, the mechanism, however, was not investigated. Perhaps, the voids between Ta-MBG particles allow fibrinogen adsorption on glass surfaces, and 4 nm-sized mesopores enhance water absorption. Nonetheless, fibrin fibers appear to embrace Ta-MBG surfaces to lay the foundation of a blood clot. It can be concluded that Ta-MBGs provide a stable and physiologically acceptable surface for blood clot formation, while Arista^TM^ offers an unstable barrier to the blood flow. The stability of the Ta-MBGs in the wet environments, such as bleeding, was also reported previously due to their negative zeta potentials [53].

Hemostasis is physiologically achieved by vascular constriction, platelet plug formation, and coagulation cascade [26]. Arista^TM^ enhances hemostasis by inducing platelet plug formation, which is conditional to the patient-specific conditions [37]. Hypothermia and coagulopathy lead to defective platelet adhesion affecting platelet plug formation, hence complicating hemostasis [37]. The hypothermic and coagulopathic conditions of the animals in the present study are one of the potential causes of the inferior hemostatic capability of Arista^TM^. Conversely, Ta-MBGs, in addition to enhancing platelet plug formation, can also activate the intrinsic pathway of coagulation due to their negatively-charged surfaces, which is reflected as the superior hemostatic capability of these materials.

Ta-free MBGs increased PTT and showed the highest parenchymal hemorrhage and necrosis extent post-intervention as seen through histopathological analysis (Table 4). Ta’s absence in the undoped MBG can be a reason for 0Ta’s hemorrhage and necrosis induction. After the Ta-free MBG, Arista^TM^ caused the second highest hemorrhage and necrosis. 5Ta is the only composition reducing the severity of the hemorrhage and necrosis after its application (Table 4); this means that 5Ta can mitigate the postoperative bleeding complications. Previous in vitro analysis also showed the 5 mol% Ta in the Si-Ca-P BG network optimized the composition for hemostatic purposes [53]. The present study also indicates that Ta’s presence (and its content) influences the coagulation capabilities of Ta-MBGs. Still, the role of Ta in enhancing hemostatic processes is not fully understood. The histopathological analysis shows a similar influence of Arista^TM^ and 1Ta on hemorrhage and necrosis extent, post-intervention, while 5Ta proved to be the only composition reducing the severity of hemorrhage and necrosis after application. Histopathological examination also showed that all the tested interventions increased the clot adherence after their respective applications. But 5Ta was the only sample that helped to achieve a minimal adherent clot (+) in a profuse bleeding condition where no adherent clot (−) was seen before the intervention; all the other interventions helped to increase the adherent clot (+++) on the initial foundation laid down by the physiological adherent clot (+) formed before the application of the intervention.

Ta-MBGs provided better handling characteristics in the bleeding surgical field than Arista^TM^. It was noticed that Arista^TM^ is very lightweight and tends to pillow when applied, spreading particulate matter into the environment; it tends to get washed away in profuse bleeding because it couldn’t hold itself onto the flowing blood. Arista^TM^ is a purified starch powder, and generally, such powders have a density of 1.5 gm/cm^3^ [59]; Arista^TM^ has been reported as a low mass, high volume, and low-density product [60]. On the other hand, the presence of a metallic element, Ta, with a density of 16.65 gm/cm^3^ in Ta-MBGs provides a powder with higher density, which helps them to settle on the profuse bleeding. This facilitates Ta-MBG powders absorbing on the fluid component of the blood more efficiently. As noted in the previous study, Ta-MBGs possess nanopores of about 4 nm size giving these BGs high surface areas [52]. It is proposed that efficient absorption of fluids by Ta-MBGs led to reduced bleeding times than Arista^TM^. The literature also mentions the problem of swelling with Arista^TM^ in enclosed wounds, which can cause ischemia to the associated tissue [32, 37]. BGs, on the other hand, do not swell; rather, they degrade after coming in contact with the body fluids [1, 2], and hence are free of swelling problems.

Arista^TM^ particles are spherical with their diameters ranging from 30 to 100 μm [61] (average diameter of 76 μm) [36]. Conversely, Ta-MBGs, are much smaller (average diameter of 1 μm) and show irregularity in shape and size and display smooth and irregular surfaces. Spherical hemostatic powders, such as Arista^TM^, present uniform packing patterns devoid of voids in between as compared to irregularly shaped particles [36]. The voids presented by irregular particles (here, Ta-MBGs) allow blood to penetrate while spherical morphologies (Arista^TM^) swell by absorbing the fluid in their porous surface, creating a tamponade above the bleeding site [36]. As previously detailed, the pressure built up by the flowing blood usually dislodges the tamponades [36]. It is postulated that in the current study, Ta-MBGs provided voids for fluid absorption, while Arista^TM^ provided a physical barrier that got dislodged, leading to longer bleeding times. This observation is another potential reason for Arista^TM^’s inferior and Ta-MBG’s superior hemostatic performance here.

In hospitals, severe bleeding cases are conventionally managed with the administration of either fresh frozen plasma, cryoprecipitate, platelets, or fibrinogen concentrate to reverse the dilutional coagulopathy [22, 54]. Among the various management modalities for dilutional coagulopathy following exsanguination, the fibrinogen concentrate offers multiple advantages [54], but is very expensive. Okerberg et al. [62] found the cost to be between $680 and 800 per gram. They further added that the cost should be reduced to $414 per gram to be economically competitive to cryoprecipitate [62]. Hemostatic dressings were initially developed for military settings but are now widely used in hospitals because they reduce healthcare costs [27, 28]. The BGs can be fabricated relatively easily at a low price using cheap inorganic precursors, while fibrinogen concentrate is derived from fresh blood. Geurts et al. [28] confirmed the cost-effectiveness of a BG (S53P4) based one-stage treatment of chronic osteomyelitis than a two-stage treatment using gentamicin-loaded PMMA. They reported the unit price for one gram of BG as €89 (~$105) [28]. The reduced cost offered by the BGs can help reduce the financial burden on the healthcare system, providers, and recipients [27, 28]. Additionally, fibrinogen can cause infections and needs special storage conditions, while BGs are not burdened by these disadvantages [17].

The present study has limitations. A trauma patient with severe bleeding conditions undergoes damage control surgery, resuscitation in the intensive care unit, and planned return to the operating room, which takes considerable time [20]. The current study represents a short resuscitation and observation time after the treatment; the timeframe used here does not fully represent the clinical situation. The present research lacks blood loss data. A comparison of the blood loss data among the experimental groups would have signified the clinical application of the developed Ta-MBGs. Euthanizing pigs right after the surgery is also a drawback; the animals after the surgery could have been evaluated to elucidate on Ta-MBGs toxicity and inflammatory potential. Additionally, lower fibrinogen concentrations in the current study could cease the bleeding within 10 min and fibrinogen concentrations are directly related to the clot density and adhesion [24]. The role of the Ta-MBGs in clot density and adhesion should also be explored in future experiments.

## Conclusions

Despite the lower fibrinogen levels at the end of the procedure, Ta-MBGs could stop bleeding within 10 min of their application. Conversely, the gauze and Arista^TM^ allowed bleeding for up to 45 min. It is proposed that additional features of negatively-charged surfaces, availability of a stable matrix, and better handling properties shown by Ta-MBGs provide them superior hemostatic properties than Arista^TM^. Furthermore, the irregular shape and higher density of Ta-MBGs give them an excellent matrix to absorb the fluid component of the blood than Arista^TM^. 5Ta compositions displayed outstanding hemostatic performances and the capacity to improve severe bleeding complications such as necrosis. Fibrinogen therapy is beneficial for dilutional coagulopathy conditions post-hemorrhage but is a costly alternative. The development of economically prepared MBG-based hemostats, such as presented here, can provide technologically advanced mechanisms to stop bleeding and reduce the healthcare system’s financial burden. Additionally, these platforms can be further iterated to load biologics to achieve targeted tissue applications.
